# Editorial: Genomics and phenomics of crop wild relatives (CWRs) for crop improvement

**DOI:** 10.3389/fpls.2023.1221601

**Published:** 2023-06-02

**Authors:** Parimalan Rangan, Kanakasabapathi Pradheep, Sunil Archak, Petr Smýkal, Robert Henry

**Affiliations:** ^1^ Division of Genomic Resources, Indian Council of Agricultural Research (ICAR)-National Bureau of Plant Genetic Resources, New Delhi, India; ^2^ Queensland Alliance for Agriculture and Food Innovation, The University of Queensland, St Lucia, QLD, Australia; ^3^ Indian Council of Agricultural Research (ICAR)-National Bureau of Plant Genetic Resources (NBPGR)-Regional Station, Thrissur, India; ^4^ Department of Botany, Faculty of Science, Palacký University, Olomouc, Czechia

**Keywords:** crop wild relative (CWR), genomics, phenomics, domestication cost, candidate gene analysis, *de novo* domestication

Crop wild relatives (CWRs) exhibit a close relationship with domesticated crops (agri-horticultural, medicinal and aromatic, ornamental, and forestry species) and form a part of the crop’s gene pool with potential for gene exchange. A large number of CWRs are potential donors but receive less attention than domesticated crops. CWRs have also suffered from genetic erosion resulting in a severe loss of genetic diversity (Maxted et al., 2006; von Wettberg et al., 2020). The factors that drive this loss in genetic diversity have been categorized into remote drivers and proximate drivers that act on the evolutionary forces: mutation, migration/gene flow, genetic drift, and selection (Khoury et al., 2022).

In this Research Topic, Trainin et al. documented from an anatomical perspective, the evolutionary forces involved in the selection of the non-foliar photosynthesis trait in the stems of *Prunus arabica* (Olivier) Meikle (a CWR of almond) when compared to the commercial almond (*P. dulcis* (Mill.) D. A. Webb). The stems of *P. arabica* favor stem photosynthesis for additional carbon gain through multiple strategies. Higher stem photosynthesis in *P. arabica* than in *P. dulcis* is attributed to selective anatomical features such as the presence of a high density of sunken stomata in their stems, a chloroplast-rich mesophyll-like parenchymatous cell layer, higher chlorophyll content, better chlorophyll fluorescence and quenching parameters, and its ability to efficiently regulate water loss at elevated temperatures.

Zhu et al. identified the molecular mechanism of an R2R3-MYB transcription factor gene underlying a drought tolerance trait, *VyMYB24*, isolated from a drought-tolerant grapevine wild relative– *Vitis yanshanesis* J. X. Chen. This gene was identified as a nuclear protein that was significantly upregulated during drought to impart drought stress tolerance and functionally validated through the genetic transformation of tobacco. Transgenic plants overexpressing the *VyMYB24* gene were phenotypically dwarfed with a lower leaf area, reduced flower size, and seed weight. The role of VyMYB24 in regulating the gibberellin biosynthetic pathway was studied.

Du et al. reported the introgression of stripe rust and Fusarium head blight resistance genes from *Leymus mollis* (Trin.) Pilg. (2n = 4x = 28, NsNsXmXm) into wheat cv. 7182 through an alien disomic substitution line, M862 (2n = 6x = 42, 21II), using an inter-specific octoploid hybrid *Tritileymus* (2n = 8x = 56, AABBDDNsNs or AABBDDXmXm). Cytogenetic studies indicate that the disomic substitution line (M862) carried 4Ns in place of 4D chromosomes. A few structural variations in chromosomes 1A, 1D, 2B, and 5A were also reported and the transcriptome analysis identified the genes and Ns-specific markers associated with resistance genes that had utility in wheat improvement programs.

With the availability of genomics tools, whole genome sequence information, and a reference set for the desired trait representing the complete range of trait variation, it is very possible to identify the candidate genes underlying the trait variation. Gowda et al. used a genome-wide association approach to mine the candidate genes associated with the straw silica content trait in the germplasm of the rice progenitor, *Oryza nivara* S.D.Sharma & Shastry, representing the variability of 5-16% silica content among a set of 258 accessions. This study led to the identification of the candidate genes, an ATP-binding cassette (ABC) transporter, casparian thickenings, multi-drug and toxin extrusion (MATE) protein, F-box, and MYB-transcription factors.

An understanding of the factors underlying genetic diversity loss will help identify ways to reverse genetic diversity losses and support the introgression of desirable traits into domesticated ones. Though gene introgression from wild to crops is possible, it is mostly untapped due to the deleterious effects of linkage drag reducing genetic gain and selection efficiency and, thus, gene introgression is considered to be the ‘cost of domestication’ (Moyers et al., 2018). Besides linkage drag, pulses and oilseeds do not yield higher genetic gain due to low variation. Hence, to create variation and thereby achieve acceptable genetic gain, mutation breeding has been the method of choice for these two crop groups in earlier decades. Singh et al. underscore the difficulties in achieving higher genetic gain with special reference to legume crops. Their research provides an overview of the key strategic approaches such as broadening the genetic base through alien introgression from CWRs. In addition, integration of the strategic approaches with modern tools, such as genetic engineering, genome editing, and speed breeding, along with present-day omics tools will potentially bring a paradigm shift in legume breeding programs to achieve higher genetic gain with minimal costs.

An understanding of the genomics of linkage drag (Huang et al., 2023) would help balance the domestication cost. Besides, CWRs play an important role in understanding crop geography and hence its origin, as proposed by Vavilov (1935). Barazani et al. discuss the history of olive cultivation using archaeological evidence that documented the presence of plant remains of wild olive, *Olea europaea* L. subsp. *europaea* var. *sylvestris* (Mill.) Lehr during the Middle Pleistocene (roughly 780,000 years ago) in the Southern Levant region. Selection pressure on this wild form gave rise to the present-day cultivated olives, *Olea europaea* subsp. *europaea* var. *europaea*. This warrants further genetic studies among the naturally grown olive populations with traditional landraces and modern cultivars to understand the loci being subjected to selection pressure in the domestication process. With the proven strength of genome editing tools in the creation of *de novo* domesticated crop plants in the CWRs of model crop plants such as rice and tomato (Li et al., 2018; Yu et al., 2021), a broader understanding of the CWRs in the -omics era will potentially help accelerate crop improvement.

## Author contributions

Author PR wrote the first draft and all authors have contributed to finalize the manuscript. All authors contributed to the article and approved the submitted version.
